# Emergency laparotomy risk assessment: An audit of South Australian hospitals

**DOI:** 10.1016/j.sipas.2023.100225

**Published:** 2023-11-24

**Authors:** Joseph N. Hewitt, Thomas J. Milton, Octavia Tz-Shane Lee, Joshua Tinnion, Antonio Barbaro, Katarina Foley, Ishraq Murshed, Nick Georges, Rippan Shukla, Cameron Main, Christopher Dobbins, Markus I. Trochsler

**Affiliations:** a Discipline of Surgery, The University of Adelaide, The Queen Elizabeth Hospital, South Australia, Australia; b Department of Surgery, Royal Adelaide Hospital, South Australia, Australia; c Adelaide Medical School, The University of Adelaide, South Australia, Australia; d Department of Surgery, Mount Gambier Hospital, South Australia, Australia; e Department of Surgery, Berri Hospital, South Australia, Australia; f Department of Anaesthesia, Royal Adelaide Hospital, South Australia, Australia

**Keywords:** General surgery, Emergency laparotomy, Risk assessment

## Abstract

**Background:**

Emergency laparotomy (EL) is associated with high mortality rates and is performed on a heterogenous patient population. Pre-operative risk assessment is one tool which can assist with EL patient care. We aimed to characterise rates of pre-operative risk assessment for EL patients in South Australia.

**Methods:**

A retrospective audit of all patients undergoing EL over one year in six participating hospitals in South Australia was undertaken. Patient demographics, operation details, risk assessments (e.g. NELA, POSSUM, ACS-NSQIP) and outcomes were recorded.

**Results:**

422 ELs were audited. Preoperative risk assessments were recorded for 42 (10 %) operations. The 30-day mortality rate was 9 %. There was no difference in mortality rates for patients with or without a risk assessment documented. Hospital participation in the Australia and New Zealand Emergency Laparotomy Audit (ANZELA) was associated with increased rates of risk assessment. Increasing patient age and then presence of certain comorbidities were also associated with increased rates of risk assessment.

**Conclusions:**

This audit shows poor uptake of recommendations for preoperative risk assessment in EL patients in South Australia. Comparable mortality rates to previously published Australian and international data are demonstrated. Factors associated with increased risk assessment rates are identified and are relevant to future quality improvement activities.

## Introduction

Emergency laparotomy (EL) is a common but high-risk procedure performed by general surgeons on a highly heterogenous group of patients. Estimates of mortality vary from 7.1 % in-hospital mortality in the Australian context [1] to 9.2 % 30-day mortality in UK estimates [2]. Mortality is higher still for the elderly patient [1,3].

Assessment of risk prior to contemplated EL is clearly important for multiple reasons. Prior knowledge of risk may influence decisions by patients, families, surgeons, anaesthetists and intensivists on whether to proceed with surgery at all, the urgency of surgery and its appropriate setting, the appropriate seniority of staff to be involved, the suitable post-operative destination and the expected outcome. Preoperative risk assessment documentation has been shown previously in a prospective study to be associated with lower mortality after operation [4]. Various risk assessment tools are easily available online such as the UK's National Emergency Laparotomy Audit (NELA) risk calculator, the Portsmouth-Physiological and Operative Severity Score for the enumeration of Mortality and Morbidity (P-POSSUM) and the American College of Surgeons National Surgical Quality Improvement Program (ACS-NSQIP) risk calculator.

Despite listing as a key performance indicator in the Australia and New Zealand Emergency Laparotomy Audit-Quality Improvement (ANZELA-QI), a joint effort of the Royal Australasian College of Surgeons (RACS) and the Australia New Zealand College of Anaesthetists (ANZCA), less than half of all patients undergoing EL in an Australian sample have a pre-operative calculation of risk documented [1]. This contrasts with UK data from NELA where risk assessment rates exceed 85 % [2]. Little is known about why clinicians choose not to use available risk assessment tools although some limited survey-based data is available [5]. This is despite preoperative risk assessment being a simple and quick process which can potentially help direct the most appropriate allocation of scarce resources [6]. The state of preoperative risk assessment in Australia is not known outside of a limited number of hospitals which subscribe to ANZELA-QI. It may be that an ‘observer effect’ increases rates of risk assessment in these hospitals due to its listing as a key performance indicator on the audit. Rates of risk assessment at non-ANZELA hospitals may be even lower again. Accordingly, we carried out a retrospective audit aiming to characterise rates of EL risk assessment documentation in South Australian public hospitals and factors which may influence risk assessment documentation. We hypothesised that some of these factors might be patient related such as age, comorbid status or urgency of operation; or system related such as time of admission or presentation to hospital, method of arrival at hospital, and consultant presence.

## Methods

A retrospective audit of all ELs carried out in two metropolitan and four regional public hospitals in South Australia in 2021 was performed. The study is reported in accordance with STROBE checklist for observational cohort studies [7].

### Hospitals

The Royal Adelaide Hospital (RAH) and the Queen Elizabeth Hospital (QEH) are both major tertiary hospitals in metropolitan Adelaide. Mount Gambier Hospital (MGH), Port Augusta Hospital (PAH), Riverland General Hospital (RGH) and Whyalla Hospital (WH) are all located in regional South Australia, serving local populations of less than 30,000 people. At the time of interest in this study the RAH and MGH were the only South Australian hospitals participating in ANZELA. Data are presented deidentified by hospital, labelled metropolitan hospital 1 and 2 and regional hospital 1 through 4.

### Participants

All patients undergoing EL in 2021 in participating hospitals were included for analysis. EL was defined as any open abdominal general surgical procedure undertaken on a non-elective basis; for example: laparotomy and adhesiolysis, colonic or small bowel resection, open appendicectomy by midline incision, repair of perforated peptic ulcer etcetera. Procedures converted from a laparoscopic to open approach were included. Procedures where extension of a laparoscopic port site or mini-laparotomy were undertaken only for extracorporeal anastomosis or specimen retrieval were excluded. Unplanned returns to theatre were included but planned returns (e.g. for closure of laparostomy) were excluded. Patients undergoing trauma, vascular or gynaecological procedures were excluded.

Lists of patients undergoing an emergency procedure in 2021 in each hospital were generated from operating theatre management software. Patient records were reviewed to determine if each case was included or excluded in the audit. Data collection took place from March to December of 2022.

### Variables

The primary endpoint was the rate of documentation of the use of a pre-operative risk assessment tool (e.g. NELA, POSSUM, NSQIP etc.). Secondary endpoints included mortality in hospital, at 30-days and 90-days post operation, complications (defined using the Clavien-Dindo system), time of presentation to hospital and time of operation, post operative disposition (ward, HDU, ICU), post operative length of stay, destination on hospital discharge and readmission within 30 days. Time of presentation to hospital and time of operation were dichotomised into inside and outside business hours which was defined as 8am to 5pm.

Patient demographic details were collected, along with selected comorbidities, details of the operation performed and timing, consultant presence in theatre, pre-operative location (ward, ED, HDU, ICU), pre-morbid accommodation, preoperative antiplatelet or anticoagulant drugs taken and whether preoperative computed tomography was performed.

Where pre-operative risk assessment was not undertaken, a NELA score was calculated retrospectively.

### Statistical analysis

The significance level was set at 0.05. Statistical analysis was undertaken using Jamovi (The Jamovi Project, Sydney, Australia). Statistical tests including chi-square, students *t*, and linear regression were undertaken as appropriate to the data in question. Anticipating that some patients would have multiple operations, each operation was analysed as a separate episode of an opportunity for a risk assessment to occur, however in calculating mortality and outcomes and reporting patient demographics each patient was only included once.

### Ethical approval

Ethical approval was sought from the Central Adelaide Local Health Network Human Research Ethics Committee (CALHN HREC). The study was deemed exempt from full review as a quality improvement activity. After study completion the CALHN HREC provided publication ethical approval (reference 16,081).

## Results

### Patient demographics

422 ELs were performed in participating hospitals during 2021. 36 patients underwent two or three ELs (including returns to theatre). The median age was 68 years. The youngest patient was 6 years old and the oldest was 97. Three patients under the age of 18 were included, all of whom were operated on in regional hospitals. Patient demographic characteristics are displayed in Table 1 below, split by hospital location. Patients operated in metropolitan hospitals were more likely to be older (*p* < 0.001), have atrial fibrillation (*p*= 0.043), be on antiplatelet or anticoagulant medication (*p*= 0.006), and have a greater post-operative length of stay (*p*= 0.005).

**Table 1:** Patient demographics.

	Metropolitan hospitals (*n*= 332)	Regional hospitals (*n*= 49)	p value
Median age [IQR] (years)	69 [55 – 79]	58 [45 – 73]	< 0.001
Male sex	42% (142)	57% (28)	0.061
Comorbidities			
Ischaemic heart disease	12% (41)	4% (2)	0.088
Atrial fibrillation	11% (38)	2% (1)	0.043
COPD	10% (32)	14% (7)	0.316
Diabetes mellitus	21% (70)	16% (8)	0.441
Hypertension	41% (136)	27% (13)	0.053
Dementia	2% (8)	4% (2)	0.494
No pre-operative antiplatelets or anticoagulation	74% (276)	92% (46)	0.006
Malignancy found at operation	17% (59)	14% (7)	0.547
Arrived by interhospital transfer	27% (88)	16% (8)	0.123
Median post-operative length of stay [IQR] (days)	8 [6 – 13]	5 [3 – 8]	0.005

138 (33 %) operations involved a colectomy, 77 (18 %) involved adhesiolysis and 78 (18 %) involved small bowel resection. Only 16 (4 %) involved patch or repair of a gastric or duodenal ulcer.

### Risk assessment and mortality rates

A risk assessment was recorded for 42 laparotomies. Rates of risk assessment documentation and mortality at hospital discharge, 30-days and 90-days are shown in Table 2, broken down by hospital. Where risk assessment was documented it almost exclusively consisted of a NELA score although two patients had a NSQIP and two patients had a P-POSSUM score calculated. There was a significant difference in risk assessment documentation rates between hospitals participating in ANZELA and those that did not (15 % vs 2 %, *p* < 0.001).

**Table 2:** Risk assessment and mortality rates by hospital.

Hospital	Metro 1	Metro 2	Regional 1	Regional 2	Regional 3	Regional 4	Total
Patients	221	111	2	16	23	8	381
ELs	245	127	2	17	23	8	422
Preoperative risk assessment documented	28 (11%)	3 (2%)	0 (0%)	0 (0%)	11 (48%)	0 (0%)	42 (10%)
In-hospital mortality	23 (10%)	10 (9%)	0 (0%)	0 (0%)	1 (4%)	0 (0%)	34 (9%)
30-day mortality	26 (12%)	9 (8%)	0 (0%)	0 (0%)	1 (4%)	0 (0%)	36 (9%)
90-day mortality	31 (14%)	14 (13%)	0 (0%)	0 (0%)	1 (4%)	0 (0%)	46 (12%)

There was no difference in mortality rates at hospital discharge (*p*= 0.184), 30-days (*p*= 0.113), or 90-days (*p*= 0.293) for patients with or without a risk assessment documented. Similarly, there was no association between risk assessment documentation and readmission within 30 days (*p*= 0.410). Mortality at 30-days was associated with increased age (64 vs 70, *p*= 0.029) as was mortality at 90-days (64 vs 71, *p*= 0.006). The NELA mean predicted mortality was significantly higher in patients who died at 30-days compared to those who survived (30.9 % % vs 6.4 %, *p* < 0.001). Higher NELA mean predicted mortality was associated with post-operative admission to intensive care or a high dependency ward (*p* < 0.001).

The expected overall mortality at 30-days in this study based on NELA scores was 8.45 % compared to the observed overall 30-day mortality of 9.45 %.

There was a significant difference in mean age between patients who had a documented risk assessment and those who did not (74 vs 63, *p* = < 0.001). Significant differences in risk assessment rates were present also for patients with selected comorbidities. This is displayed in Table 3.

**Table 3:** Risk assessment rates for patients with and without selected comorbidities.

Comorbidity	Rate of risk assessment for patients with condition	Rate of risk assessment for patients without condition	p value
Ischaemic heart disease	10% (35 / 338)	14% (6 / 43)	0.473
Atrial fibrillation	21% (8 / 39)	10% (33 / 342)	0.038
COPD	21% (8 / 39)	10% (33 / 342)	0.038
Diabetes mellitus	17% (13 / 78)	9% (28 / 303)	0.059
Hypertension	15% (22 / 149)	8% (19 / 232)	0.043
Dementia	30% (3 / 10)	10% (38 / 371)	0.047

There was no difference in risk assessment documentation rates between operation booking urgency categories (*p*= 0.525).

220 operations (52 %) were conducted within business hours and 202 (48 %) out of business hours. There was no association between risk assessment and operations conducted out of business hours (*p*= 0.493).

185 operations (44 %) were performed on patients presenting to hospital in business hours and 237 (56 %) outside of business hours. There was no association between risk assessment and patients presenting to hospital outside of business hours (*p*= 0.396).

### Interhospital transfer

Arrival by interhospital transfer compared to any other method had no effect on patient mortality at hospital discharge (*p*= 0.304), 30-days (*p*= 0.513) or 90-days (*p*= 0.477) in metropolitan hospitals.

Similarly, arrival by interhospital transfer compared to any other method had no effect on patient mortality at hospital discharge (*p*= 0.632), 30-days (*p*= 0.632) or 90-days (*p*= 0.632) in regional hospitals.

Arrival by interhospital transfer had no effect on risk assessment rates in metropolitan (*p*= 0.576) or regional hospitals (*p*= 0.074).

### Consultant presence

Consultant surgeons were present in theatre for 360 (85 %) ELs. Consultants were the primary surgeon at all 50 (100 %) ELs in regional hospitals. Consultants were the primary surgeon for 169 (40 %) ELs overall. Surgical fellows were the primary surgeon for 183 (43 %) and registrars were the primary surgeons for 70 (17 %). There was no association between consultant presence and in hospital (*p*= 0.473), 30-day (*p*= 0.607) or 90-day (*p*= 0.314) mortality. Likewise, there was no association between consultant presence and risk assessment rates (*p*= 0.319).

## Discussion

This study demonstrated an overall rate of 10 % risk assessment for emergency laparotomy in participating South Australian hospitals. Rates of documentation of preoperative risk assessment are low in this study, especially considering the recommendation for universal risk assessment within ANZELA, and this represents an aspect of EL perioperative care ripe for quality improvement. This has been extensively noted previously [6,8] and some suggestions for increasing risk assessment rates have been made such as mandated risk scoring at the time of theatre booking for laparotomy [4]. There is significant heterogeneity between risk assessment rates between hospitals and this study identified hospital participation in ANZELA as one factor associated with this. Although ANZELA hospitals performed much of the risk assessment in this study there was still a large difference between risk assessment rates between those two hospitals, with regional hospital 3 risk assessing 48 % of ELs compared to metropolitan hospital 1′s 11 %. This difference is potentially explained by a discrepancy in size and staff numbers between the hospitals. Regional hospital 3 is staffed by four general surgeons with one SET registrar whereas general surgery at metropolitan hospital 1 includes five subspecialty units, each with multiple consultants, a SET registrar, and numerous junior medical staff. Likewise, the size of the anaesthetic departments in each hospital is vastly different. Promoting a culture of adherence to risk assessment recommendations may be easier in a smaller setting with less staff to educate and inform of those practices. In contrast, small departments may be subject to greater resource limitations which might lead to increased difficulties with implementing risk assessment or changing practices. Our data cannot confirm either suggestion and further research is needed to understand factors leading to differing rates of risk assessment.

The accuracy and utility of NELA scores was also demonstrated with a significant association between higher NELA predicted mortality and actual mortality and an observed to expected mortality ratio close to one. Where risk assessment is performed in South Australian hospitals, clinicians show a strong preference for NELA score use over other possible risk assessment tools.

Increasing patient age and the presence of atrial fibrillation, COPD, hypertension, and dementia were associated with increased rates of preoperative risk assessment. This suggests clinicians carry out risk assessment more often in cases they already perceive as being high risk rather than regarding risk assessment as a more universal screening tool. Universal risk assessment should be implemented given the benefits to informed consent even in low-risk patients and given the potential to identify high risk patients earlier and intervene to alter outcomes for those patients. Universal risk assessment should also be adopted on the basis of the Perth Emergency Laparotomy Audit (PELA) identifying that “30-day mortality in those who had a prospectively documented risk assessment was lower than those in whom it was retrospectively calculated (5.2 % and 9.5 %, respectively)” [4] and the UK's National Emergency Laparotomy Audit (NELA) showing that those without a risk assessment have similar mortality rates to those assessed as high risk [3]. A mortality difference between those who were and were not risk assessed was not observed in this study though the study is likely underpowered to detect this due to the low overall numbers of risk assessment. Further light could be shed on any possible association between risk assessment and outcomes by large scale prospective audits such as ANZELA.

The relatively recent uptake of electronic medical records in South Australia may represent an opportunity to streamline or automate risk assessment. The majority of data required to calculate a NELA score are physiological or biochemical parameters which could be found within the electronic medical record. Electronic medical records or theatre booking software could incorporate prompts to undertake risk assessment also.

Contrary to expectations, urgency of operation booking and time of presentation to hospital and time of operation had no effect on risk assessment rates. This suggests lack of opportunity to perform risk assessment due to time pressure, urgency or competing demands is not a strong factor influencing lack of risk assessment. Again contrary to expectations, there was no difference in mortality rates between patients arriving by interhospital transfer and by other methods. This may be influenced by the inclusion of transfers from other metropolitan public or private hospitals to the tertiary hospitals in our dataset in this category, as these transfers would be expected to take less time than transfers from regional hospitals to metropolitan hospitals.

Overall 30-day mortality rates and post operative length of stay we identified in this study are comparable to previously published Australian data [9,10]. 30-day mortality in regional hospitals was low (2 %) and comparable to that identified by a previous audit focussed on three of these hospitals, the Rural Emergency Laparotomy Audit (RELA) [11].

A comparison of this study's outcomes with those of ANZELA, PELA, RELA and NELA is shown below in Table 4. A small but nevertheless encouraging improvement in risk assessment rates will be noted between RELA and this study. Higher rates of risk assessment noted in PELA are likely due to the prospective nature of the study and its inclusion as part of the bundle of care associated with the audit. Higher rates of risk assessment noted by the retrospective ANZELA and NELA audits however are likely due to an observer effect of auditing risk assessment. Risk assessment rates have increased over the years that NELA has been running in the UK [2].

**Table 4:** Comparison of studies of emergency laparotomy.

	ANZELA [12]	NELA [2]	PELA [4]	RELA [11]	This study
Patient characteristics					
Number of ELs	3178	22,132	198	51	422
Median age (years)	66	*	66	62	68
Median expected 30-day mortality (%)	*	*	5.6	3.5	3.2
Procedure					
Colectomy (%)	*	32	29	29	33
Adhesiolysis (%)	*	18	18	22	18
Small bowel resection (%)	*	15	18	20	18
Outcomes					
Preoperative documentation of risk assessment (%)	52	87	69	6	10
Mean length of stay (days)	13	10§	10	10	12
30-day post operative mortality (%)	6‡	9†	7	0	9

⁎ not available.

§ mean.

† in-hospital mortality.

‡ in-hospital, 30 day.

This study has several limitations. Retrospective data collection and retrospective calculation of NELA scores introduce elements of bias. Additionally, not all South Australian hospitals participated. No private hospitals participated. As private hospitals have previously been estimated to undertake up to 11 % of all ELs in Australia [9] this limits the generalisability of our findings to the public sector only. Data on the seniority of anaesthetic staff present were not readily available as this is not routinely recorded in operative notes in the hospitals sampled. It is likely that all patients operated in regional hospitals were cared for by a fully qualified specialist anaesthetist or rural generalist anaesthetist however this could not be confirmed. This data is collected prospectively by ANZELA and is an area for further investigation in our study.

This study has characterised the adherence to recommendations for risk assessment prior to emergency laparotomy in participating South Australian hospitals. Although heterogenous, rates of risk assessment in general are low. In the UK, NELA auditing adherence to these recommendations has resulted in a reduction in 30-day mortality from 13 % to 9 % over eight years of the project [2]. This study also demonstrates a preference for NELA scores as the most commonly used risk assessment tool in South Australia and further quality improvement activity should focus on improving rates of its use.

## Data availability

Data supporting this study are available on request to the corresponding author, JNH. Data are not publicly available to preserve patient privacy.

## CRediT authorship contribution statement

**Joseph N. Hewitt:** Conceptualization, Data curation, Formal analysis, Investigation, Methodology, Project administration, Writing – original draft, Writing – review & editing. **Thomas J. Milton:** Data curation, Writing – review & editing. **Octavia Tz-Shane Lee:** Data curation, Writing – review & editing. **Joshua Tinnion:** Data curation, Writing – review & editing. **Antonio Barbaro:** Data curation, Writing – review & editing. **Katarina Foley:** Data curation, Writing – review & editing. **Ishraq Murshed:** Data curation, Writing – review & editing. **Nick Georges:** Data curation, Writing – review & editing. **Rippan Shukla:** Data curation, Writing – review & editing. **Cameron Main:** Data curation, Writing – review & editing, Supervision. **Christopher Dobbins:** Conceptualization, Data curation, Supervision, Writing – review & editing. **Markus I. Trochsler:** Conceptualization, Data curation, Writing – review & editing.

## Declaration of Competing Interest

The authors declare the following financial interests/personal relationships which may be considered as potential competing interests: Joseph Hewitt reports financial support was provided by the Australian Government. Christopher Dobbins reports a relationship with Surgery in Practice and Science that includes: editorial board membership. The other authors declare that they have no known competing financial interests or personal relationships that could have appeared to influence the work reported in this paper.
